# CARE Registry: An update & plans to further expand the meaningful inclusion of Asian American, Native Hawaiian, and Pacific Islander populations in Alzheimer's disease and related dementias research

**DOI:** 10.1002/alz70860_098473

**Published:** 2025-12-23

**Authors:** Van Ta Park, Janice Y. Tsoh, Bora Nam, Marian Tzuang, Daren Huang, Gabriel Fara‐On, Cati Brown Johnson, Thomas J. Hoffmann, Oanh L. Meyer, Arnab Mukherjea, Christy Nishita, Dolores Gallagher‐Thompson, Ladson Hinton, Vaatausili Tofaeono, Poki'i Balaz, Justina P Tavana, Quyen Q Tiet, Joyce R. Javier, Quyen Vuong, Hye‐Won Shin, David Choi, Joshua D Grill

**Affiliations:** ^1^ University of California San Francisco School of Nursing, San Francisco, CA, USA; ^2^ Asian American Research Center on Health (ARCH), University of California San Francisco, San Francisco, CA, USA; ^3^ Multi‐Ethnic Health Equity Research Center, University of California, San Francisco, San Francisco, CA, USA; ^4^ University of California San Francisco School of Medicine, San Francisco, CA, USA; ^5^ Evaluation Sciences Unit, Division of Primary Care and Population Health, Stanford School of Medicine, Stanford, CA, USA; ^6^ University of California San Francisco School of Nursing, Office of Research, San Francisco, CA, USA; ^7^ University of California San Francisco, Department of Epidemiology and Biostatistics, San Francisco, CA, USA; ^8^ University of California, Davis School of Medicine, Sacramento, CA, USA; ^9^ California State University, East Bay, Hayward, CA, USA; ^10^ Center on Aging, University of Hawaii, Honolulu, HI, USA; ^11^ Department of Psychiatry and Behavioral Sciences, Stanford University School of Medicine, Stanford, CA, USA; ^12^ University of California at Davis Betty Irene Moore School of Nursing, Davis, CA, USA; ^13^ Department of Neurology. University of California, Davis, Sacramento, CA, USA; ^14^ American Samoa Community Cancer Coalition, Pago Pago, American Samoa; ^15^ Kokua Kalihi Valley Comprehensive Family Services, Honolulu, HI, USA; ^16^ Brigham Young University, Provo, UT, USA; ^17^ California School of Professional Psychology at Alliant International University, San Francisco, CA, USA; ^18^ Department of Health Systems Science, Kaiser Permanente Bernard J. Tyson School of Medicine, Pasadena, CA, USA; ^19^ International Children Assistance Network, Milpitas, CA, USA; ^20^ Somang Society, Cypress, CA, USA; ^21^ Institute for Memory Impairments and Neurological Disorders, University of California, Irvine, Irvine, CA, USA; ^22^ YE Media Group, Los Angeles, CA, USA

## Abstract

The Collaborative Approach for AANHPI Research and Education (CARE) registry aims to address underrepresentation of Asian American, Native Hawaiian, and Pacific Islander (AANHPI) populations in aging, Alzheimer's disease and related dementias (ADRD) and caregiving research. Guided by community‐based participatory research principles, all aspects of the CARE registry are propelled by fostering trust and partnerships between academic and community stakeholders. These partnerships enable culturally‐tailored engagement strategies to advance recruitment, retention, and inclusion of AANHPI participants in research. Participants enroll by completing a survey online, in‐person, or by phone, covering socio‐demographics, health, caregiving status, and other information. The survey is available in a growing list of languages including English, Chinese (Simplified/Traditional), Hindi, Japanese, Korean, Samoan, Tagalog, and Vietnamese. Since CARE's launch in October 2020, 10,485 AANHPI adults have enrolled (as of January 13, 2025). The mean age is 54.3 years (standard deviation (SD)=17.9; range: 18‐101), with 34.8% (*n* = 3,657) older adults (≥65 years old). Approximately 14% (*n* = 1,419) of the participants reported having ADRD symptoms. Among the 1,072 participants who were caregivers, 40.4% reported caring for someone with ADRD. Most (83.1%) CARE participants were foreign‐born, with 50.0% speaking limited English. The major cultural groups were Vietnamese (38.2%), Chinese (30.0%), Korean (14.8%), Asian Indian (5.5%), Filipino (5.0%), Native Hawaiians and Pacific Islanders (NHPI) (3.4%), and Japanese (3.2%).

CARE is also actively engaging researchers to support their study recruitment of AANHPI participants. Of the 130 recruitment referral requests received, 64 (65.3%) were aging‐related, including 49 (38%) ADRD or dementia caregiving studies. Since January 2021, CARE has made over 14,900 referrals to 51 completed studies, with 7,060 CARE participants referred to at least one study. Originally with recruitment primarily in California, CARE is now expanding nationally to enroll an additional 10,000 participants. This effort includes offering additional languages and forming strategic partnerships to target areas with significant populations of AANHPI older adults and targeted outreach efforts to AANHPI groups currently underrepresented in CARE (e.g., Filipino, Asian Indian, NHPI). CARE seeks to advance recruitment and retention science by exploring effective strategies for engaging and retaining AANHPI participants and assessing the registry's impact on research enrollment outcomes.

